# Feasibility of progesterone treatment for ischaemic stroke

**DOI:** 10.1177/0271678X15616782

**Published:** 2015-11-10

**Authors:** Claire L Gibson, Philip M Bath

**Affiliations:** 1 Department of Neuroscience, Psychology and Behaviour, University of Leicester, Leicester, UK; 2 Stroke, Division of Clinical Neuroscience, University of Nottingham, Nottingham, UK

**Keywords:** Steroids, cerebrovascular disease, drug trials, focal ischaemia, acute stroke

## Abstract

Two multi-centre phase III clinical trials examining the protective potential of progesterone following traumatic brain injury have recently failed to demonstrate any improvement in outcome. Thus, it is timely to consider how this impacts on the translational potential of progesterone treatment for ischaemic stroke. A wealth of experimental evidence supports the neuroprotective properties of progesterone, and associated metabolites, following various types of central nervous system injury. In particular, for ischaemic stroke, studies have also begun to reveal possible mechanisms of such neuroprotection. However, the results in traumatic brain injury now question whether further clinical development of progesterone for ischaemic stroke is relevant.

## Introduction

Progesterone is a gonadal steroid hormone historically associated with reproductive function. However, over the last 15–20 years we have begun to understand how progesterone, and other steroid hormones, can directly influence central nervous system (CNS) function. Such steroid hormones, including oestrogens, progesterone and androgens, modulate cognition, mood, myelination, neurogenesis, inflammation and recovery from various types of injury. Progesterone’s actions are mediated by the widespread distribution of progesterone receptors within the CNS (for review see literature^1^).

A wealth of experimental evidence supports the neuroprotective properties of progesterone following CNS injury including models of traumatic brain injury (TBI), cerebral ischaemic stroke, spinal cord injury, diabetic neuropathy, amyotrophic lateral sclerosis, neuroinflammation and neuropathic pain.^2^ Following CNS injury, progesterone has been shown to be pleiotropic acting via a multitude of mechanisms that include, but are not restricted to, affecting the processes of inflammation, oedema formation, apoptosis, excitotoxicity, dysmyelination, lipid peroxidation and oxidative stress.^3^ The experimental evidence for a protective role of progesterone is strong, particularly in the cases of TBI, ischaemic stroke and dysmyelinating disorders. Three single-centre and two multi-centre clinical trials for TBI have now been completed. However, in light of the results of such TBI clinical trials it is now timely to consider if there is any feasibility in the utility of progesterone for ischaemic stroke.

## Progesterone and TBI – the clinical perspective

Progesterone was first identified as having neuroprotective properties in the CNS by the observation that pseudopregnant female rats experienced less damage following induced TBI compared to males or normally cycling females. Such protection was shown to be dependent upon high circulating levels of progesterone.^4^ A large body of work (reviewed elsewhere^5^) has provided further evidence that progesterone treatment can provide protection following TBI. As a result, three independent small-scale, individual centre clinical trials were launched. The first randomized clinical trial of progesterone for acute TBI (ProTECT) was successfully completed in 2007 and reported that use of progesterone following TBI was well tolerated in terms of safety and resulted in a lower 30-day mortality rate compared with the placebo group.^6^ Two other clinical trials, conducted by the same investigators,^7,8^ investigated the effect of progesterone treatment on neurological outcome following TBI. The first of those trials^7^ did not report sufficient data to dichotomize outcomes into favourable and unfavourable outcomes.^9^ However, the second trial, by the same group, demonstrated improved neurological outcome in progesterone-treated TBI patients.^8^ Thus, two of these single-centre clinical trials reported favourable effects in terms of progesterone reducing mortality and improving neurological outcome following TBI and consequently two multi-centre phase III clinical trials were launched.

The first large study, progesterone for TBI, Experimental Clinical Treatment (PROTECT III) was a phase 3, randomized, double-blind, placebo-controlled clinical trial designed to determine the efficacy of early intravenous administration of progesterone versus placebo for treating patients with non-penetrating TBI caused by a blunt mechanism.^10^ Patients were recruited if they were deemed to have experienced severe, moderate-to-severe, or moderate acute TBI defined by their score on the Glasgow Coma Scale (GCS). The trial was funded by the National Institute of Neurological Disorders (NINDS) and conducted through the NINDS-funded Neurological Emergencies Treatment Trials (NETT) network. Efficacy, in terms of improvement following treatment, was assessed using the stratified dichotomy of the Extended Glasgow Outcome Scale (GOS) score assessed at six months post-injury along with identified secondary outcomes including mortality and the Disability Rating Scale score. A total of 882 of the planned sample of 1140 patients underwent randomization before the trial was stopped, at the second interim analysis, because of futility related to the primary outcome, i.e. GOS score.^10^

The second multi-centre clinical trial was aimed at examining the possible effect of progesterone following severe TBI. The study of a neuroprotective agent, progesterone, in severe TBI (SYNAPSE) completed enrolment of all the intended 1195 participants.^11^ SYNAPSE, sponsored by BHR Pharma, was a multinational (21 countries), prospective, double-blind, parallel-group trial in which patients with severe TBI were randomly assigned to intravenous progesterone and placebo. Again, efficacy was determined by an improvement in the primary outcome measure of GOS score at six months post-injury. Although the trial did complete participant recruitment, no benefit of progesterone treatment was observed as compared with placebo in terms of GOS score and mortality.^11^

**Figure 1.**
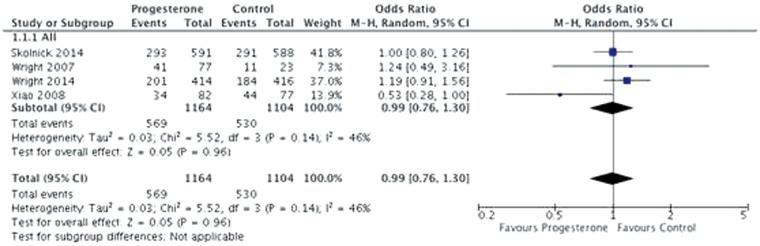
Forrest plot of the effect of progesterone on poor outcome (dead, vegetative state or severe disability) in patients with traumatic brain injury.

When the data from the five clinical trials are aggregated, there is no evidence that progesterone modifies functional outcome, assessed as the proportion of patients with a poor outcome (GOS dead, vegetative state or severe disability) (Table 1, Figure 1). Similarly, progesterone did not alter death (Table 1). Nevertheless, heterogeneity was present for both analyses (I^2 ^> 40%) although the cause of this remains unexplained.

**Table 1.** Effect of progesterone on outcome in patients with traumatic brain injury. Data are numbers, odds ratio (95% confidence intervals) and heterogeneity. Analysis using Mantel-Haenszel random effects model.

	Progesterone (n/N)	Control (n/N)	Odds ratio	Heterogeneity
Poor functional outcome	569/1164	530/1104	0.99 (0.76, 1.30), *p* = 0.96	*p* = 0.14, I^2 ^= 46%
Death	224/1222	201/154	0.90 (0.59, 1.36), *p* = 0.61	*p* = 0.04, I^2 ^= 60%

n = number of participants with stated outcome; N = total number of participants receiving treatment.

## Progesterone and TBI – comparing experimental and clinical design

Unfortunately, the failure of these two large multi-centre clinical trials for TBI (i.e. PROTECT III and SYNAPSE) adds to the growing number of neutral or inconclusive clinical trials trying to identify a successful treatment for TBI. The failure of these two clinical trials was not predicted from either the experimental studies or the previous single-centre clinical trials that had occurred. However, important differences exist between the design of preclinical studies, single-centre clinical trials and multi-centre clinical trials. Undoubtedly experimental studies are conducted with much rigour and homogeneity and it can be argued that similar homogeneity occurs in single-centre clinical trials. However, a completely different design is used when multi-centre trials are involved, and although heterogeneity is a feature of these it may not be present in preclinical experimental studies.

The failure of a potential treatment, such as progesterone, to improve outcome following TBI may be a consequence of the complexity and variability of the injury. However, the measures used to assess both extent of injury at time of admission and outcome following treatment (or placebo) were not sensitive to the complexity of injury. In both large multi-centre clinical trials, patients were assessed for the extent of TBI using the GCS, which only assesses signs and does not assess the underlying extent of pathological damage.^12^ Thus, it was not possible to assess the heterogeneity of pathological damage in patients and group according to extent of damage. In addition to the limited assessment at the time of recruitment into the trial, the outcome measures used were also relatively insensitive. Both trials used the GOS score as the primary outcome measure along with mortality as a secondary outcome. Yet almost all experimental studies assessing the effectiveness of progesterone treatment following TBI used extent of pathological damage as the primary outcome.^13^ Although more comprehensive tests of neurological and behavioural function do exist, such as the NIH toolbox, which have been validated in both animal and human studies these are more expensive (due to training required) and time consuming to incorporate into clinical trials. The ability to detect efficacy following any intervention probably requires assessment of relevant biomarkers that would clearly assist diagnosis and outcome assessment. Although potential biomarkers have been identified in experimental and clinical TBI studies,^14,15^ they are still lacking appropriate validation and implementation.^16^ Ideally, clinical studies would contain a variety of measures in order to better characterize TBI and facilitate individualized treatment.^17^ Given the failure of these two clinical trials, a re-review of the TBI translational research strategy is warranted.

Along with TBI, progesterone has also shown great promise as a neuroprotective agent in experimental stroke studies. It may not be surprising that an agent, such as progesterone, is effective in experimental TBI studies and experimental stroke studies as the two share many pathological features including excitotoxicity, oxidative stress, apoptosis and inflammation. However, the sources of primary injury are distinct in that TBI produces shear mechanical forces that can act to disrupt cellular membrane integrity, whereas ischaemic stroke produces ionic perturbations which trigger a specific and complex biochemical cascade. However, in terms of translating experimental treatments into clinical practice one might argue that researchers within the stroke field have spent the last 10 years closely scrutinizing the design of preclinical (and clinical) studies thus, it may be that progesterone offers more hope for the treatment of ischaemic stroke. Guidelines have been produced by the Stroke Therapy Academic Industry Roundtable^18^ and subsequently updated^19^ in order to ensure that comprehensive experimental studies are completed prior to considering clinical trial. Although of course such guidelines do not necessarily guarantee compliance and a large number of experimental stroke studies do not necessarily adhere to them.

## Progesterone as a neuroprotective strategy following ischaemic stroke

Like TBI, there is substantive experimental evidence showing progesterone and related metabolites are protective following experimental ischaemic stroke in rats and mice.^20–22^ In addition, limited studies have shown effectiveness in aged^23–25^ and hypertensive animals^26^ although extensive studies examining the effect of progesterone neuroprotection in animals with stroke-specific morbidities (i.e. diabetes, obesity, hypertension) in both genders are currently lacking. Such protective effects of progesterone have largely been demonstrated by reporting decreased infarct volume and improvements in functional outcome following administration of progesterone following stroke onset.^13^ However, experimental studies tend not to report factors such as survival following treatment although this is a key safety outcome measure in clinical trials.

In a recent individual data meta-analysis examining progesterone treatment for ischaemic stroke we showed that progesterone treatment was significantly associated with an increase in the incidence of death.^27^ Although not necessarily reported as significant in individual studies, either because studies didn’t report death or were too small to allow detailed analysis, it did become significant when all data from published studies were combined. The most apparent effect was seen in aged animals compared to younger animals and in females compared to males. It’s likely that sex plays an influence in effectiveness of treatments and it may be that progesterone, for example, is a treatment worthy of consideration only for males experiencing ischaemic stroke. The importance of gender-specific research and the influence of gender on treatment effectiveness were highlighted recently at the 2014 Academic Emergency Medicine Consensus Conference. In addition, it still remains to be determined in experimental studies what, if any, interaction progesterone has with tissue plasminogen activator (T-PA) and whether it has any detrimental effect in spontaneous intracerebral haemorrhage. Although one recent study has shown that progesterone administration may actually extend the time window of T-PA administration and reduce haemorrhagic transformation.^28^ Both of these are important translational aspects to address as they will impact significantly on the feasibility of progesterone treatment for stroke in a clinical setting. It’s also worth noting that an effective treatment may not necessarily be progesterone per se but a derivative/metabolite or direct receptor agonist.

However, stroke research, like TBI, has a poor success rate for delivering experimental therapies into effective clinical treatments. In the last 15–20 years over 1000 experimental compounds have shown efficacy in experimental stroke studies with almost 200 progressing to clinical trial.^29^ Yet the only pharmacological therapies currently in use for ischaemic stroke are those with low efficacy but administered to the majority of patients (e.g. aspirin) or thrombolysis with alteplase which is only applicable to approximately 15% stroke patients.^30^ What the results from the TBI clinical studies, and other failed trials, demonstrate is the need for the design of experimental and clinical studies to more closely match.^31^ Progesterone has never been explored in a multi-centre animal trial and it could be argued that this critical step should have been performed before progressing onto multi-centre clinical trials and actually should form an essential part of the validation process for any prospective treatment.

## Conclusions

Stroke is a heterogeneous disorder and it is unlikely that we are going to identify one treatment that ‘cures all’ – it may be that progesterone treatment is effective but only for a subset of patients and/or effective only as part of a multi-modal therapy. However, in light of the two failed phase III TBI clinical trials it is timely to consider the translational questions that still need to be investigated in preclinical studies of stroke before considering progressing to a clinical trial. Important questions include the potential interaction of progesterone with T-PA, possible gender-specific effects of progesterone, development of progesterone receptor-specific targets and the effectiveness of progesterone in a multi-centre preclinical study.
